# The Effect of Perineural Adjuvants on Superficial Parasternal Intercostal Plane Blocks in Cardiac Surgery: A Triple-Blinded Randomized Controlled Feasibility Trial

**DOI:** 10.7759/cureus.75967

**Published:** 2024-12-18

**Authors:** Rawad I Hamzi, Scott R Coleman, Salvador Pena, Joni K Evans, Heidi Whiteside, Bryan E Marchant, Karuna Puttur Rajkumar, Wessley McKnight, Shelby Harris, Nataya S Disher, Anusha N Samant, Rohesh J Fernando

**Affiliations:** 1 Department of Anesthesiology, Wake Forest School of Medicine, Winston Salem, USA; 2 Department of Biostatistics and Data Science, Wake Forest School of Medicine, Winston Salem, USA

**Keywords:** adjuvant, analgesia, cardiac surgery, median sternotomy, pain management, pecto-intercostal fascial plane block, pifb, regional anesthesia, spip, superficial parasternal intercostal plane

## Abstract

Background and aim

The study aimed to investigate the effect of adding perineural adjuvants, clonidine and dexamethasone, to local anesthetic in Superficial Parasternal Intercostal Plane (SPIP) blocks. It was designed as a prospective, randomized, triple-blinded, feasibility trial, conducted at a single-center university hospital. The participants included adult patients who were undergoing cardiac surgery via median sternotomy.

Methodology

Following skin closure, patients were randomized to receive either SPIP with 0.25% bupivacaine and 2.5 mcg/mL epinephrine (control group, *n *= 12) or SPIP with 0.25% bupivacaine, 2.5 mcg/mL epinephrine, 1.67 mcg/mL clonidine, and 0.1 mg/mL preservative-free dexamethasone (adjuvant group, *n *= 8).

Results

No significant difference was found between the adjuvant and control groups for the primary outcome of the area under the curve of longitudinal pain scores with incentive spirometry use measured at four timepoints during the first 24 hours following surgery (96.5 ± 58.4 vs. 94.3 ± 39.6, *P *= 0.93, respectively). Additionally, no difference was found for secondary outcomes, including opioid consumption in morphine milligram equivalents (120, interquartile range [IQR] 93-150 vs. 120, IQR 82-158; *P *= 0.88), time to extubation (232.02 ± 68.69 vs. 276.82 ± 78.61 min, *P *= 0.25), intensive care unit length of stay (31.4 vs. 38.2 hours, *P *= 0.64), or pain satisfaction scores between the adjuvant and control groups (*P *= 0.46), respectively. The adjuvant group demonstrated higher incentive spirometry volumes at the six-hour postoperative timepoint (1333.33 ± 857.97 mL vs. 525.00 ± 338.56 mL, *P *= 0.003), with a trend toward a difference at 12 and 18 hours (*P *= 0.24 and *P *= 0.10, respectively).

Conclusions

The addition of perineural adjuvants to SPIP was not associated with any difference in pain scores within the first 24 hours after cardiac surgery. Given the small nature of this feasibility study, further investigation is warranted.

## Introduction

Pain is common after cardiac surgery, especially in the initial postoperative days [1]. As many as three-fourths of patients report moderate or greater levels of pain [2]. Achieving adequate postoperative analgesia is paramount, as failure is associated with adverse outcomes such as altered hemodynamics, increased myocardial consumption, impaired pulmonary mechanics, and increased length of stay [3]. Furthermore, a small study found that moderate to severe postoperative pain persisted in over 50% of patients at 30 days, highlighting the need to identify interventions that may mitigate this pain course [4]. While pain regimens have historically relied heavily on opioids, opioids are not completely benign, potentially placing postoperative cardiac surgery patients at risk for continued use of opioids after surgery. In light of the opioid epidemic, multimodal non-opioid medications and regional anesthesia have gained popularity to stymie opioid consumption [3].

Regional anesthesia for postoperative pain management in cardiac surgery is not a recent phenomenon, as epidural, paravertebral, and parasternal field blocks have been used in the past. Fascial Plane Blocks (FPBs) have several advantages over neuraxial and deep block techniques because they are technically easier to perform and do not share the same concern for anticoagulation during cardiac bypass [3]. Therefore, FPBs have become a common approach to blocking afferent sensory innervation of the chest wall for patients undergoing cardiothoracic surgery involving sternotomy. In particular, the Superficial Parasternal Intercostal Plane (SPIP) block is one such approach that anesthetizes the anterior cutaneous branches of the second through the sixth intercostal nerves, which innervate the sternum and overlying soft tissue, by injection of local anesthetic in the fascial plane between the pectoralis major and internal intercostal muscles [5,6]. The SPIP Block has been demonstrated to reduce postoperative resting and dynamic pain scores, postoperative opioid consumption, time to extubation, duration of intensive care unit (ICU) stay, and incidence of postoperative nausea and vomiting (PONV) in patients following cardiac surgery [5,6].

Perineural adjuvants are commonly used in peripheral nerve blocks (PNBs) to extend the duration of analgesia, but they remain relatively understudied in FPB. Specifically, the glucocorticoid steroid dexamethasone, alpha-2 adrenergic receptor agonists dexmedetomidine and clonidine, and the opioid receptor agonists nalbuphine and buprenorphine have been used to varying degrees of success for extending the duration of PNB [7-11]. Extending the analgesic duration of a single-shot SPIP Block is highly clinically relevant, given that catheter-based techniques require deploying foreign bodies close to the surgical field of the sternum bilaterally, which could create an additional nidus for infection and become at risk of displacement.

The purpose of this randomized, triple-blinded, prospective, feasibility study was to compare the level and duration of postoperative analgesia provided by SPIP Block when performed with local anesthetic solution with or without perineural adjuvants clonidine and dexamethasone in patients following cardiac surgery involving sternotomy. We hypothesized that the patients receiving SPIP Block with multiple local anesthetic adjuvants, bupivacaine and epinephrine, clonidine, and dexamethasone would have lower dynamic pain scores over the first 24 hours compared to those receiving SPIP Block with just bupivacaine and epinephrine.

## Materials and methods

Study design

This was a randomized, triple-blinded, prospective, feasibility trial that took place after approval from our Institutional Review Board (IRB00090669). Investigational New Drug Exemption was obtained beforehand from the US Food and Drug Administration for the use of the perineural adjuvants under the existing IND exemption 125810. Written informed consent was obtained from all study participants before randomization, and the trial was registered on clinicaltrials.gov before enrollment (NCT05676814). Patients were enrolled from March 27, 2023, to June 23, 2023.

Selection criteria

Inclusion Criteria

Adults between 18 and 90 years of age undergoing cardiac surgery involving median sternotomy at Atrium Health Wake Forest Baptist Medical Center who consented to participate and did not meet exclusion criteria were recruited for participation in the study. 

Exclusion Criteria

Patients with any contraindications to regional anesthesia, such as history of allergy to amide local anesthetics or any of the perineural adjuvants; existing neurologic deficit in the chest wall; those who were deemed unlikely to be extubated within the first six hours (judgement made prior to randomization), or those that did remain intubated at the six-hour time point after block placement (assessed after surgery); weight under 50 kg; undergoing emergency surgical procedures or urgent return to the operating room; active endocarditis or mediastinitis; moderate or severe right ventricular dysfunction after cardiopulmonary bypass; reliance on mechanical circulatory support devices, such as intra-aortic balloon pump or ventricular assist device; localized or systemic infection; chronic use of high-dose opioid analgesics (defined as daily use greater than 60 oral morphine milligram equivalents [OMME] for over one month before surgery) or those whose pain was anticipated to be difficult to control based on preoperative medications and/or comorbidities (e.g., amphetamine use); as well as those who were pregnant, were excluded from participation. Any patients who refused enrollment or withdrew their consent were excluded, as were patients whose cardiac surgeon requested exclusion. Any patient who was enrolled in another investigational trial that did not allow co-enrollment was excluded.

Sample size and randomization

A sample size of 20 patients was chosen since this was a feasibility study, and to the best of our knowledge, no prior studies have examined the effect of these specific adjuvants at these doses for this specific block. Patients were anesthetized and monitored per our usual cardiac anesthesiology practice with general anesthesia. At the end of cardiopulmonary bypass, patients were reassessed for the previously mentioned exclusion criteria before being randomized to one of the two treatment arms: SPIP Block with bupivacaine and epinephrine (control group) or SPIP Block with bupivacaine, epinephrine, clonidine, and dexamethasone (adjuvant group). Randomization occurred in blocks using SealedEnvelope and was done by the investigational pharmacy that prepared the study medication. All surgical and anesthesia personnel, as well as patients, were blinded to their treatment arm assignment, as were all research personnel who collected outcome data, and critical care staff caring for the patients in the postoperative period. The SPIP Block was performed after skin closure and before transport from the operating room to the ICU. 

SPIP Block technique

After skin closure, bilateral SPIP Block was placed by a faculty anesthesiologist with experience in the performance of ultrasound-guided regional anesthesia. Starting at the clavicle, the ribs were counted while moving the probe caudad. A longitudinal, parasagittal view of the costal cartilage 1-2 cm lateral to the sternum at the level of the fourth or fifth ribs or between the fifth and sixth ribs was obtained. A 10 cm 20-gauge STIMUPLEX Ultra 360 needle (B. Braun, Tokyo, Japan) was inserted in the cephalad direction in the plane between the pectoralis major and internal intercostal muscles, to obtain cephalad spread along the plane over multiple costal cartilage levels, as described previously [12]. If adequate cephalad spread was not achieved, the procedure was repeated at a higher interspace. The SPIP Block was performed bilaterally using a volume of 30 mL of local anesthetic solution per side. 

Patients randomized to the control arm received bilateral SPIP Block with a total of 60 mL of 0.25% bupivacaine with 2.5 mcg/mL epinephrine (1:400,000 dilution), while those in the experimental arm received bilateral SPIP Block with a total of 60 mL of 0.25% bupivacaine with 2.5 mcg/mL epinephrine (1:400,000 dilution), 1.67 mcg/mL clonidine, and 0.1 mg/mL preservative-free dexamethasone. Following placement of bilateral SPIP Block, patients were intubated to the ICU with a propofol infusion for sedation, as per institutional protocol for routine postoperative care.

As is our current practice, patients were managed with ICU nursing-administered titrated boluses of intravenous fentanyl for analgesia before extubation based upon signs of nociception, including hypertension, tachypnea, facial grimace, and motor agitation. Patients had as-needed doses of oxycodone ordered for analgesia after extubation unless they had a contraindication or allergy, in which case a suitable alternative at equianalgesic doses was ordered.

Measurements

Upon arrival and check-in to the preoperative holding room on the day of surgery, patients were educated on the appropriate use of incentive spirometry (IS) and asked to rate their baseline verbal pain score both at rest and with IS on the 11-point numerical rating scale (NRS: 0-10, where 0 represents no pain and 10 represents the worst imaginable pain).

The time of block placement was documented as the zero-hour time point for measurements. As described above, to collect data for the primary outcome, research staff elicited patient-reported NRS pain scores with the use of IS in triplicate at 6, 12, 18, and 24 hours post-block placement. Data were also collected for secondary outcome measures, including, but not limited to, eliciting resting NRS pain scores at 6, 12, 18, and 24 hours after block placement; measuring IS volumes in triplicate at 6, 12, 18, and 24 hours; measuring the time after block placement to first opioid administration; time to extubation; duration of ICU admission; calculating incidence of nausea and vomiting based on the administration of the antiemetics ondansetron, metoclopramide, and promethazine; calculating incidence of delirium based on bedside ICU nurse documentation of Confusion Assessment Method in the ICU (CAM-ICU) score of 3 or greater (0-2 represents no delirium, 3-5 represents mild to moderate delirium, and 6-7 represents severe delirium) at any point in the first 24 hours after block placement; and measuring patient-reported NRS satisfaction with the analgesic regimen at the 24-hour point (0 represents completely unsatisfied and 10 represents completely satisfied). The cumulative 24-hour opioid consumption was calculated by converting all opioids to OMME to allow for appropriate comparison.

Statistical methods

Basic patient characteristics were described using mean and standard deviation or median and IQR for continuous variables or number and percent for categorical variables. Continuous variables were compared between randomized arms using a Wilcoxon rank-sum test, and categorical variables were compared using Fisher’s exact test. The usage of opioids and other medications, as well as ICU duration and the pain management satisfaction score, were similarly described and compared.

The primary outcome was defined to be the area under the curve (AUC) defined by the longitudinal pain scores (baseline, 6, 12, 18, and 24 hours post-surgery) with IS use. The AUC calculation required a score at each time point. Four patients (two in each group) had missing pain scores a total of six times; these were imputed using a mixed-effects modeling framework that allowed the use of all available data to estimate means at each time point. These estimates were used as the imputed values for the six missing scores. Because the distributions of pain scores were highly non-normal, a negative binomial mixed model was fit to the available data (using the SAS GLIMMIX procedure). Additionally, because three scores were recorded at each time point, both the average and maximum values of those three were calculated, and AUC was calculated using both sets of values. AUC was normally distributed and was compared between randomized arms using a t-test; analysis of covariance was used to adjust for age, gender, BMI, surgeon, and procedure type (CABG vs. other).

A negative binomial mixed model was used to assess the difference between randomized arms in the trajectory of pain scores over time, beginning with six hours post-surgery, adjusting for the baseline score and accounting for repeated measurements. Similar mixed models assessed the difference between randomized arms in the trajectory of resting pain scores over time (recorded once per time point) and IS volumes (average of three measurements at each time point).

## Results

**Figure 1:**
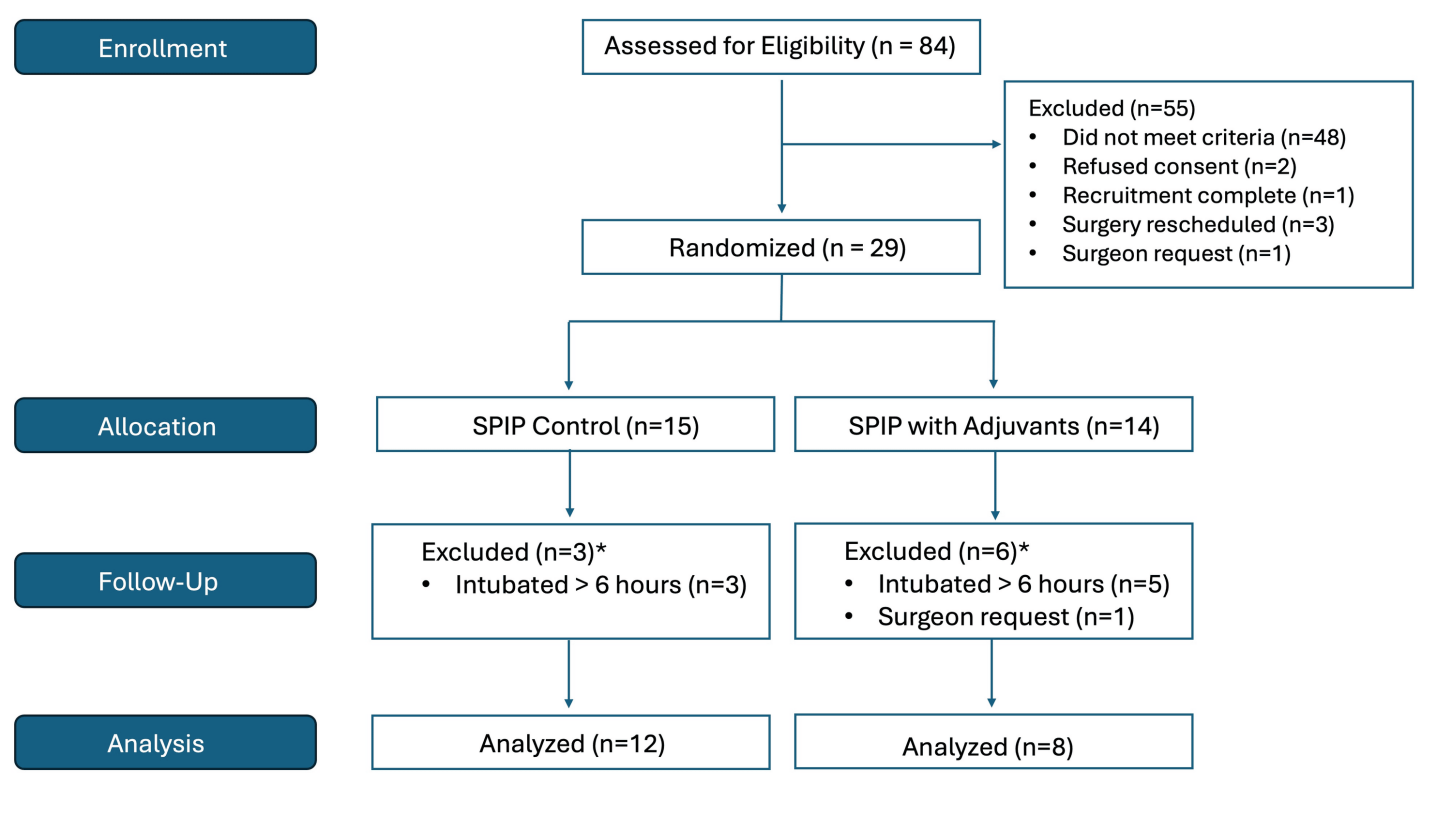
CONSORT diagram. *These patients were excluded based on a priori criteria. SPIP, Superficial Parasternal Intercostal Plane; CONSORT, Consolidated Standards of Reporting Trials

Eighty-four patients were assessed between March 2023 and June 2023. After 55 patients were excluded, the remaining 29 patients were randomized to the SPIP Block control group (*n *= 15) or the SPIP Block adjuvant group (*n *= 14). After randomization, nine patients were excluded based on a priori criteria because of failure to extubate within six hours of surgery (*n* = 8) and surgeon request (*n *= 1). Of the remaining 20 patients, 12 were in the SPIP Block control group and eight were in the SPIP Block adjuvant group (Figure 1). The basic characteristics of these patients by randomized arm are shown in Table 1. There were no significant differences in patient characteristics between the two groups. 

**Table 1:** Patient characteristics. Continuous variables were compared using the Wilcoxon rank-sum test, and categorical variables were compared using Fisher’s exact test. BMI, body mass index; SD, standard deviation; IQR, interquartile range; *n*, number; SPIP, Superficial Parasternal Intercostal Plane; surgeon, each attending cardiac surgeon whose patients received a block as part of this study was assigned a number to determine if there were any differences based on attending surgeon

Characteristic	Total (*n* = 20)	SPIP control (*n* = 12)	SPIP adjuvant (*n* = 8)	*P*-value
Age (years), median (IQR)	65.5 (58.5-71.5)	69.5 (63-72.5)	60.5 (56-65.5)	0.0584
Age (years), mean ± SD	65.2 ± 8.9	68.4 ± 7.6	60.3 ± 8.8	
Gender, *n* (%)				0.9999
Female	3 (15.0%)	2 (16.7%)	1 (12.5%)	
Male	17 (85.0%)	10 (83.3%)	7 (87.5%)	
Race, *n* (%)				0.9999
Black	1 ( 5.0%)	1 ( 8.3%)	0 ( 0.0%)	
White	19 (95.0%)	11 (91.7%)	8 (100%)	
Height (m), median (IQR)	1.78 (1.75-1.83)	1.78 (1.75-1.84)	1.77 (1.75-1.82)	0.5334
Weight (kg), median (IQR)	92.7 (78.9-100.50)	83.3 (76.2-93.7)	99.5 (92.2-101.8)	0.1228
BMI, median (IQR)	27.5 (26.4-32.3)	27.0 (24.9-29.4)	31.4 (27.4-32.7)	0.076
ASA, *n* (%)				0.9999
III	3 (15.0%)	2 (16.7%)	1 (12.5%)	
IV	17 (85.0%)	10 (83.3%)	7 (87.5%)	
Procedure type, *n* (%)				0.9999
CABG	16 (80.0%)	10 (83.3%)	6 (75.0%)	
Other	4 (20.0%)	2 (16.7%)	2 (25.0%)	
Surgeon, *n* (%)				0.91
1	6 (30.0%)	3 (25.0%)	3 (37.5%)	
2	1 ( 5.0%)	1 ( 8.3%)	0 ( 0.0%)	
3	9 (45.0%)	6 (50.0%)	3 (37.5%)	
4	4 (20.0%)	2 (16.7%)	2 (25.0%)	

AUC is summarized in Table 2. There were no significant differences between arms. None of the selected covariates were significantly related to the outcomes, and their inclusion in the model did not affect the significance of the difference between arms (*P *= 0.79 for an average of three and *P *= 0.80 for a maximum of three).

**Table 2:** Area under the curve (AUC) for pain scores with incentive spirometry. ^§^AUC units were obtained by plotting numerical rating scale scores over 24 hours. ^*^P-values are from t-tests. SD, standard deviation; IQR, interquartile range; SPIP, Superficial Parasternal Intercostal Plane; AUC, area under the curve

AUC		Total (*n* = 20)	SPIP standard (*n *= 12)	SPIP adjuvant (*n *= 8)	*P*-value*
AUC units^§^ using an average of three scores					0.9265
Mean ± SD		95.2 ± 46.5	94.3 ± 39.6	96.5 ± 58.4	
Median (IQR)		97.9 (67.8-132.0)	97.9 (80.3-117.5)	107 (39.0-140.5)	
AUC units^§^ using a maximum of three scores					0.7117
Mean ± SD		102.7 ± 47.2	99.2 ± 42.4	108.0 ± 56.1	
Median (IQR)		105.2 (71.4-144.0)	102.2 (84.9-127.5)	115.5 (54.0-153.0)	

**Figure 2:**
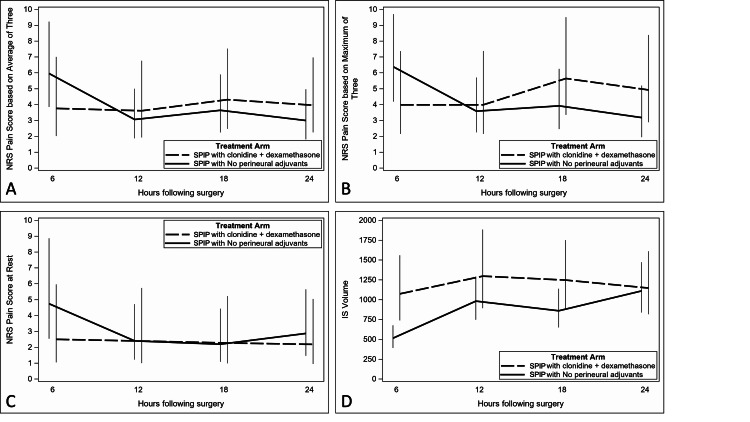
Pain scores during incentive spirometry. Pain scores measured in triplicate during incentive spirometry are shown based on (A) averages and (B) maximum scores. (C) Pain scores at rest. (D) The trajectory of incentive spirometry volumes (in mL). SPIP, Superficial Parasternal Intercostal Plane; NRS, numerical rating scale (scale of 0-10, where 0 represents no pain and 10 represents the worst imaginable pain).

The baseline pain scores for the primary outcome were nearly zero in both groups (0.38 ± 1.06 vs. 0.08 ± 0.29 in the adjuvant and control groups, respectively). Figure 2A shows the least squares means (LSMeans) with 95% confidence intervals estimated from the negative binomial mixed model for the average of three scores, and Figure 2B shows the same for the maximum of three scores. For the average scores, there was no significant arm-by-time interaction (*P *= 0.12) and there was no difference overall between arms (*P *= 0.92), but there were differences between time points (*P *= 0.013). For the maximum scores, there was no significant interaction between treatment arms and time (*P *= 0.076), and no overall difference between the arms (*P* = 0.64), nor between the time points (*P* = 0.11)

As with the AUC, a second model was fit to adjust for age, gender, BMI, surgeon, and procedure type (CABG vs. other). None of these variables were significantly related to the outcomes, and their inclusion in the model did not affect the significance of the difference between arms.

For pain at rest, there was no significant interaction between treatment arms and time (*P* = 0.50), and no overall difference between the arms (*P* = 0.59) or between the time points (*P* = 0.08). Figure 2C shows the LSMeans with 95% confidence intervals. After adjusting for age, gender, BMI, surgeon, and procedure type (CABG vs. other), there was no meaningful difference in those three test results. However, age and procedure type were significantly related to the pain at rest scores.

Regarding IS volumes, baseline values were 3427.08 ± 743.27 mL for the adjuvant group and 2750.00 ± 823.43 mL for the control group and were not statistically different (*P* =0.078). The LSMeans with 95% confidence intervals for IS are shown in Figure 2D. Overall, there was a significant arm-by-time interaction (*P* = 0.035). The six-hour IS volume in the control group was significantly lower than the volumes in the control group at other time points, but none of those other volumes were different. Among those who received adjuvants, there were no differences in IS volumes between any pair of times. The randomized arms only differed at six hours post-surgery (*P* = 0.003). After adjusting for age, gender, BMI, surgeon, and procedure type (CABG vs. other), there was no significant arm-by-time interaction (*P* = 0.08), and none of the variables were related to volume. There was no overall difference by arms (*P* = 0.27), but there were significant differences over time (*P *< 0.001).

The total opioid use in each group is summarized in Table 3. There were no significant differences between the groups. Table 4 summarizes the use of other medications in each group, as well as ICU duration and the pain management satisfaction score. None of the differences were significant. Time to extubation was also not significantly different (232.02 ± 68.69 vs. 276.82 ± 78.61 minutes, *P* = 0.25) between adjuvant and control arms, respectively. Adverse events were recorded as an exploratory outcome, but no patients suffered sternal wound infection, sternal hematoma, cardiac injury, injury to the internal mammary artery, pneumothorax, local anesthetic toxicity, or mortality.

**Table 3:** Opioid use within 24 hours after block placement. ^*^P-value for the median was based on the Wilcoxon rank-sum test. MME, morphine milligram equivalents; SD, standard deviation; IQR, interquartile range; SPIP, Superficial Parasternal Intercostal Plane

Opioid	Total (*n* = 20)	SPIP control (*n *= 12)	SPIP adjuvant (*n *= 8)	*P*-value*
Hydromorphone MME				0.4967
Mean ± SD	10.30 ± 19.4	11.00 ± 18.2	9.25 ± 22.4	
Median (IQR)	0 (0-13)	0 (0-16)	0 (0-5)	
Fentanyl MME				0.7792
Mean ± SD	54.75 ± 30.1	51.25 ± 28.9	60.00 ± 33.1	
Median (IQR)	45 (45-75)	45 (37-75)	45 (45-68)	
Oxycodone MME				0.5511
Mean ± SD	60.71 ± 24.5	57.44 ± 19.4	65.63 ± 30.7	
Median (IQR)	60 (41-75)	60.0 (45-75)	67.5 (41-75)	
Total MME				0.8772
Mean ± SD	125.8 ± 51.3	119.7 ± 48.6	134.9 ± 57.1	
Median (IQR)	120 (90-155)	120 (82-158)	120 (93-150)	

**Table 4:** Data on select secondary outcomes. Continuous variables were compared using the Wilcoxon rank-sum test, while categorical variables were compared using Fisher’s exact test. Satisfaction with the analgesic regimen at the 24-hour point was patient-reported using the numerical rating scale (0 represents completely unsatisfied and 10 represents completely satisfied). Only satisfaction scores of 4-10 are shown since no patient reported scores between 0 and 3. ICU, intensive care unit; PONV, postoperative nausea and vomiting; *n*, number; IQR, interquartile range; SPIP, Superficial Parasternal Intercostal Plane

Outcome	Total (*n *= 20)	SPIP control (*n *= 12)	SPIP adjuvant (*n* = 8)	*P*-value
ICU duration (hours), median (IQR)	32.6 (25.6-61.6)	38.2 (25.7-76.8)	31.4 (24.6-40.8)	0.6434
PONV medications, *n* (%)				0.6479
No	12 (60.0%)	8 (66.7%)	4 (50.0%)	
Yes	8 (40.0%)	4 (33.3%)	4 (50.0%)	
Acetaminophen, *n* (%)				0.5368
No	3 (15.0%)	1 ( 8.3%)	2 (25.0%)	
Yes	17 (85.0%)	11 (91.7%)	6 (75.0%)	
Other pain med, *n* (%)				0.9999
No	18 (90.0%)	11 (91.7%)	7 (87.5%)	
Yes	2 (10.0%)	1 (8.3%)	1 (12.5%)	
Satisfaction score, *n* (%)				0.4627
4	1 (5.0%)	1 (8.3%)	0 (0.0%)	
6	2 (10.0%)	2 (16.7%)	0 (0.0%)	
7	3 (15.0%)	2 (16.7%)	1 (12.5%)	
8	3 (15.0%)	2 (16.7%)	1 (12.5%)	
9	5 (25.0%)	1 (8.3%)	4 (50.0%)	
10	6 (30.0%)	4 (33.3%)	2 (25.0%)	

## Discussion

This study investigated the impact of adding the adjuvants clonidine and dexamethasone to the SPIP Block. The primary finding of this study was that the use of perineural adjuvants for the SPIP Block did not impact average or maximum pain scores over the subsequent 24 hours. Furthermore, there was no difference in the amount of opioids used, time to extubation, ICU duration, or pain satisfaction scores between groups. Interestingly, the initial set of IS volumes at six hours was statistically higher in the group that received adjuvants. While the subsequent time points did not show a significant difference, the trend at 12 and 18 hours suggested there may be an ongoing benefit in the adjuvant group. Notably, this was a smaller feasibility trial, and perhaps a larger sample size could have resulted in a statistically significant difference. Although some might attribute the difference in IS volumes to the baseline values being higher in the adjuvant group, this difference was not statistically significant, and the values were adjusted for in the statistical model. Furthermore, since the adjuvant group had IS volumes approximately twice as high as the control group at six hours, it is interesting to consider whether pain scores might have been lower if they had taken smaller tidal volumes, similar to the control group. Finally, the average pain scores at rest were also not high, which may have contributed to a lack of difference between groups.

Previous studies have demonstrated a beneficial effect of the SPIP Block compared to no block. A study by Kumar et al. randomized 40 patients to either receive a bilateral SPIP Block or no block [12]. The injections were administered at multiple sites. The local anesthetic used for the block was 0.25% ropivacaine, with a maximum total dose of 3 mg/kg. While there was no significant difference in pain scores during normal breathing at 0 and 3 hours, the SPIP Block group demonstrated significantly lower pain scores at 6 and 12 hours. In addition, the SPIP Block group required fewer rescue doses of fentanyl postoperatively. While our study showed numerically lower pain scores at six hours, this difference was not statistically significant. We speculate that a larger sample size may have shown a difference. 

Zhang et al. conducted an interesting study in which 108 patients were randomized to receive either a bilateral SPIP Block or a sham block with saline [5]. The SPIP Block was performed by injecting 0.4% ropivacaine above the second and fourth ribs on each side. It is unclear whether 20 mL was used per rib space or per side. Patients in the SPIP Block group had lower pain scores, shorter time to extubation, and reduced ICU and hospital length of stay [5].

There are notable similarities and differences between this study and those by Kumar et al. and Zhang et al. While Kumar et al. performed the SPIP Block at the end of the surgery, similar to this study, Zhang et al. performed the blocks before anesthetic induction. All three studies used injections at more than one rib space per side. Unlike the studies by Kumar et al. and Zhang et al., in which the control group received no blocks, all patients in this study received an SPIP Block with local anesthetic and epinephrine. Next, our study is unique in that we measured incentive spirometry to replicate dynamic pain during respiration, whereas Kumar and Zhang did not. Finally, our study was specifically designed to test the use of adjuvants such as clonidine and dexamethasone, which were not used in the other studies.

Another potential block for this patient population is the deep parasternal intercostal plane block (DPIP). This block is similar to the SPIP Block but places the local anesthetic in a deeper plane, between the intercostal muscles and the transversus thoracis muscle. Recently, Wong et al. randomized 86 patients to receive either DPIP with 20 mL of 0.25% levobupivacaine or placebo with normal saline in a double-blinded trial. Forty-six patients were randomized to the DPIP group, although two patients were lost to follow-up [1]. The remaining 40 patients were allocated to the placebo group. The authors noted that patients in the treatment group required fewer opioids intraoperatively, but there was no difference in opioid consumption, pain scores, or incentive spirometry values postoperatively. One concern about the DPIP, formerly known as the transversus thoracis muscle plane block, is that it requires the placement of the needle closer to the heart compared to the SPIP [13]. Furthermore, other vital structures such as the pleura and internal mammary artery may be at risk for injury. These risks, however, may be unnecessary, as Kaya et al. demonstrated that the SPIP and DPIP had similar postoperative pain scores and opioid use in a double-blinded randomized trial of 39 patients [13]. In the setting of similar efficacy, we chose to study the SPIP to increase the margin of safety.

Our group has previously demonstrated that the addition of perineural dexamethasone to long-acting local anesthetics in PNB increases the duration of analgesia, a benefit not replicated with IV dexamethasone administration, as directly assessed by sensitivity to pinprick [7,14]. Clonidine is another commonly used perineural adjuvant known to safely extend the analgesic duration of regional procedures and is well tolerated in many PNB and FPB [9,15-17]. Epinephrine has long been used as a perineural adjuvant to extend the analgesic duration of PNB by causing localized vasoconstriction, which limits the systemic uptake of local anesthetics. It also provides an added safety benefit by serving as a marker for intravascular injection [18]. While concern has been raised about the potential toxicity of perineural adjuvants when studied in vitro [19], data from in vivo studies, as well as years of experience from our group, have demonstrated the safety and tolerability of perineural epinephrine, clonidine, and dexamethasone when used in the context of safe block performance techniques, such as appropriate patient selection with exclusion of those with pre-existing neurologic deficits, awareness of injection pressure, and the addition of peripheral nerve stimulation to ultrasound guidance for blocks with motor nerve components [14,20]. We chose the dose of clonidine for this study based on prior studies from our group [16-17]. Some previous studies in healthy volunteers have used either 2 mg or 4 mg of dexamethasone for blocks [21], so our dose of 3 mg per block falls within this range. The concentration of dexamethasone is based on a prior study from our group [14].

There are several strengths to this study. First, few studies have investigated the impact of these perineural adjuvants on SPIP Blocks. Second, it was triple-blinded, meaning that the surgical and anesthesia teams, patients, and research teams collecting the data were unaware of the group assignments. Third, our study was unique in that we measured incentive spirometry, an objective measure that could be affected by pain and may be clinically important as a marker of respiratory function. Limitations of this study include the small sample size. Notably, this was a feasibility trial that required multiple measurements at several time points, including the performance of incentive spirometry in triplicate. Nevertheless, a larger trial is needed to confirm the findings of this trial. There were also several excluded patients, most of which were due to intubation for more than six hours; however, these patients were excluded based on a priori criteria, and the group assignment was not known at the time of exclusion. Furthermore, we believe this strengthened the data by making the results more comparable, as patients with prolonged intubation could have been assigned lower pain scores due to the effects of sedation. Finally, the use of patient-reported pain scores is subjective and may vary due to different levels of pain tolerance. Nevertheless, we chose to include this measure, as it is commonly reported in these types of studies.

## Conclusions

The addition of clonidine and dexamethasone to SPIP Blocks with bupivacaine and epinephrine was not associated with any significant difference in postoperative pain scores up to 24 hours after block placement. However, these adjuvants were associated with improved early incentive spirometry values. Further research with larger trials is needed to exclude a potential benefit in pain scores.
